# Sea-Sickness

**Published:** 1889-07-27

**Authors:** 


### Sea-Sickness.

We are told by medical authors that "vomiting is the forcible expulsion of the contents of the stomach through the œsophagus"; but sea-sickness is certainly something more than this, for the retching continues long after the contents of the stomach have found their way outside that organ. In these days of going to and fro on the face of the waters, the subject of sea-sickness has a greater interest for us than it had for our more "stay-at-home" forefathers. He who could discover a remedy would be famous. He would not, like the "mighty Homer," be only claimed when dead. He would receive the reward of his discovery in this life—if not in filthy lucre, assuredly in that content which we are told pervades the breast of the man who confers a benefit on his fellow-creatures. Were it not for the *mal de mer*, many who valiantly sing "I love the ocean breezes, and I love the dashing spray," would not stand fast when they arrive in touch thereof. In other words, a sea voyage to "the land of the midnight sun" or elsewhere, would become much more popular if the sight even of the "dancing waves" did not suggest immediate emetics to all not possessed of the *dura ilia messorum*. Everyone, however, is not equally susceptible to sea-sickness; neither is everyone equally susceptible at all periods. There are some few persons who enjoy complete immunity, however rough the sea may be; there are others (even naval officers) who are always affected when they first go on board ship; there is a third class who are more or less sea-sick from the time they go afloat until they disembark. Such variability must be attributed to differences of constitution, or to idiosyncrasy, just as some persons are not susceptible to the action of ipecacuanha as an emetic, while others are affected by even the smell of the drug. The "reason why" persons are not equally susceptible at all periods is to be found in their state of health, mental or bodily, at the time. If any way out of health a person is more readily affected by sea-sickness.

The number of theories which have been manufactured to account for sea-sickness is only excelled by the number of nostrums which have been brought forward as a "perfect cure." In the year 1604 Joseph Acosta wrote: "The proper and natural cause is the aire and the vapors of the sea . . . for the further we go into the sea and retyre from the land, the more we are touched and dazzled with this sickness." At a much more recent date, M. Samanes came to the conclusion, "un miasme marin est la cause essentielle du mal de mer." There is a delightful simplicity in these statements and explanations, which induces us to wish the times were primitive enough to allow us to believe them. For, on turning to modern authors who have endeavoured to explain sick-sickness, we find one writer referring it to a supplementary special sense, "the ionfunct of which is to determine the position of the head in space, and to direct and govern the œsthetiko kinetic mechanism by which is maintained the equilibrium of the body." The same author mentions that there are at least three distinct forms of sea-sickness—after which most persons will be disposed to cry, Hold! enough! Another theory is that sea-sickness depends "on an undue amount of blood in the nervous centres along the back." This may be correct, but it would be desirable to know *why* going to sea should cause the accumulation of an undue amount of blood in the back. Another writer says the stomach is deranged through "nervous influence," and that the nervous system requires time to adapt itself to the new conditions of motion. Again, all this may be very true, but we are only told what occurs, not why it occurs. When we are obliged to say a thing depends on "nervous influence," it is equivalent to a confession of ignorance, as we do not know what nervous influence is! We cannot separate nervous influence from life. An eminent author states: "All processes within the body, whether chemical, mechanical, thermic, electric, or photic, are performed by modification of the common force which produces similar phenomena in the inorganic world around us." A statement which as regards an explanation of nervous influence leaves us in exactly the same ignorance as before. Perhaps, as Tyndall observes, the mystery may resolve itself into knowledge at some future day. Hitherto, the dissection and pulling to pieces of the material body has not enhanced our conception of nervous influence.

While it is admitted that the motion of the ship is the principal cause of sea-sickness, how this motion acts can only be vaguely explained as through nervous influence. There are, however, many agencies in operation. These are unaccustomed and disagreeable sounds, sights, and smells. The conjunction of noises on board a ship is often sufficient to daze the unhabituated brain. The conglomeration of smells from oil, steam, cooking, and humanity would excite sensations of sickness in the uninitiated even on shore. The sight of others sick is the last straw to those quivering on the brink of the malady. When "around us gasp the invalids," and stewards rush about with basins, we generally resign ourselves to fate and join the majority on board. An author was quoted above as stating that "habit" enables the nervous system to adopt itself to the new conditions of motion. This is generally correct, but in some cases it is not so. In a minority of instances food sufficient to support the system cannot be taken, and dangerous exhaustion results. Or the sea-sick patient may die from syncope. Fainting and hysterical attacks are common complications of sea-sickness in delicate women. Prolonged cases of sea-sickness are also liable to terminate in dangerous fever. Sea-sickness sometimes assumes so distressing and dangerous a form as to demand the greatest care against a fatal termination. It is not always a malady which may be struggled against, or, as depicted by the poet, dear to those "who love to laugh at other's woes."

The preventatives and remedies which have been prescribed for sea-sickness are manifold. Cold brandy-and-water benefits some, but makes others worse. Champagne suits some people. Creosote, chloroform, cocaine, and a dozen other drugs, have their advocates. Others, instead of drugging, advise icebags to the spine, which may stay vomiting for a short voyage—as across the Channel. Making a strong expiratory effort—as in whistling—when sinking sensations are felt is of temporary utility. So is a tight belt with a pad to produce pressure over the stomach. A belt which may be inflated at will has been invented, by which more or less pressure may be exerted as seems requisite. Perhaps, however, the best plan is to lie flat on the broad of the back and nibble biscuits, with an occasional sip of brandy-and-water, or champagne. The fact is, however, that although there are many remedies of doubtful efficacy, there are none decidedly preventative nor curative—unless jumping overboard, as recently practised, may commend itself. But much may be done to "mitigate the ills we cannot shun." It has already been intimated that sea-sickness may be more or less violent in accordance with either the mental or bodily health of a person. It is an old and true saying that mental anxiety predisposes to every ailment under the sun, and sea-sickness is not an exception. It is not given to every man to possess the phlegm of a Socrates, who when Xantippe wound up one of her tirades with a bucket of water over her husband calmly observed that after thunder rain generally fell. But those who would avoid sea-sickness should temporarily at least cultivate a contented mind, and forget there is such "a vile anatomical organ" as the spleen in the internal economy. Similarly, it is not permitted to everyone to be always on the best of terms with a rebellious stomach, or a peccant liver. But at least a temporary truce should be established before a person conveys their troublesome organs on board ship. Sickness and vomiting may proceed, even on dry land, from a foul stomach, or from a liver out of gear. With such conditions as a basis, "waves mountain high" are not required to produce motion sufficient to excite that "nervous influence" which we are told is the *fons et origo mali*. The stomach and liver should be called to order before a person essays a sea voyage. If a person contemplating a voyage is dyspeptic or troubled with acidity, he had better consult his doctor. Or, if the fee is grudged, let him ask the wisest old woman of his acquaintance to compound him a mustard emetic. This will clean away his bile and acidity, rendering him almost proof against the *mal de mer*. It may, however, be objected that it is useless making oneself sick to avoid being so, that while the cure is desired the remedy is detested. But the condition produced by mustard does not entail that deadly feeling which accompanies sea-sickness—it is not that wonderful antidote to vanity which sea-sickness proves—and the exhibition may be rehearsed in private. When there is no dyspepsia, or acidity, or biliousness, the favourite pill—and most Britons have one—may perhaps be substituted with success. Another caution is also very requisite. If a person desires to avoid being ill on board, he should not allow his admiring friends to entertain him at dinner the night before he starts, and he should avoid those parting glasses which are quaffed with so much freedom in the saloons of ocean-going steamers. A regime as above will certainly prove much more efficacious than the nostrums with which many voyagers supply themselves. After such a preparation, with the aid of quiet and the recumbent posture at first, many, probably the majority, would escape sea-sickness altogether. Nostrums are usually of about as much utility as the "bather's manual," when thrown to a drowning man, who has only to turn up page so-and-so to ascertain how to keep himself from being suffocated.

